# TCF-3-mediated transcription of lncRNA HNF1A-AS1 targeting oncostatin M expression inhibits epithelial-mesenchymal transition via TGFβ signaling in gastroenteropancreatic neuroendocrine neoplasms

**DOI:** 10.18632/aging.203024

**Published:** 2021-05-25

**Authors:** Jingwen Xue, Jianan Bai, Qin Long, Yaling Wei, Jialing Pan, Xiaolin Li, Qiyun Tang

**Affiliations:** 1 Department of Geriatric Gastroenterology, Jiangsu People’s Hospital, Nanjing Medical University, Nanjing, China; 2 Department of Gastroenterology, Shaoxing Central Hospital, Shaoxing, China

**Keywords:** lncRNA HNF1A-AS1, gastroenteropancreatic neuroendocrine neoplasms, transcription factor 3, oncostatin M, TGFβ signaling

## Abstract

Long noncoding RNAs play key roles in several cancers, but their potential functions in gastroenteropancreatic neuroendocrine neoplasms remain to be investigated. We performed GeneChip assay to explore differentiated lncRNAs in gastric NENs and peri-cancerous tissues. The regulation of HNF1A-AS1 on biological behavior of GEP-NENs cells and *in vivo* xenograft model was confirmed by CCK8, colony formation assay, transwell, western blot and qRT-PCR. We next detected the potential transcription factors and the binding sites between them with bioinformatic analysis. qRT-PCR was performed to analyze the exact relationship between them. HNF1A-AS1 expression was decreased in gastric NENs tissues (*p* < 0.01). Over-expression of HNF1A-AS1 suppressed cellular proliferation, migration and invasion. Knockdown of transcription factor 3 inhibited the expression of HNF1A-AS1 and promoted cellular migration and invasion. Oncostatin M was identified as the downstream target of HNF1A-AS1. Inhibition of transforming growth factor-β activity inhibited HNF1A-AS1/Oncostatin M-mediated epithelial-mesenchymal transition. Our data suggest that transcription factor 3/HNF1A-AS1/Oncostatin M axis inhibits the tumorigenesis and metastasis of gastroenteropancreatic neuroendocrine neoplasms via transforming growth factor-β signaling.

## INTRODUCTION

Neuroendocrine neoplasms (NENs) are a group of human malignancies originating from the diffuse neuroendocrine system [1]. Gastroenteropancreatic neuroendocrine neoplasms (GEP-NENs) account for 60–70% of all NENs, and involve the largest hormone-producing organ of our body [2]. Epidemiological studies have shown a steady increase in incidence up to 6.98/100,000 people during the past few decades [3]. The inferior quality of living standards for individuals and more global burden to our society have been emerging due to the long course of NENs [4–7].

Long noncoding RNAs (lncRNAs) are defined as RNA transcripts with more than 200 nucleotides in length [8]. Compared with protein coding genes, lncRNAs expression may be much more tissue specific [9–11]. HNF1A-AS1, a 2455-bp transcript, was up-regulated in human colon cancer and associated with poor prognosis [12, 13], while it was down-regulated in gastric and pancreatic cancers [14–16].

Mutation of mammalian target of rapamycin (mTOR) signaling was a common pathogenic factor of GEP-NENs [14, 17, 18]. The inhibitors of mTOR signaling such as everolimus are currently standard drugs for well differentiated GEP-NENs [19]. However, the effect of everolimus is still unsatisfactory. Thus, there may be other signaling pathways which participate in the development and progression of GEP-NENs.

Epithelial-mesenchymal transition (EMT) is essential for cellular reprogramming during development [20, 21]. EMT increases the capacity of cancer cells to initiate and promotes the tumorigenesis via angiogenesis. HNF1A-AS1 inhibited malignant progression of laryngeal squamous cell carcinoma via EMT [22, 23]. LncRNAs mediated EMT was closely linked to breast cancer invasion and metastasis via transforming growth factor-β (TGF-β) signaling [24, 25]. Some EMT markers such as Snail1 were associated with the invasion and metastasis of GEP-NENs [26].

## RESULTS

### HNF1A-AS1 was down-regulated in GEP-NENs

**Figure 1:**
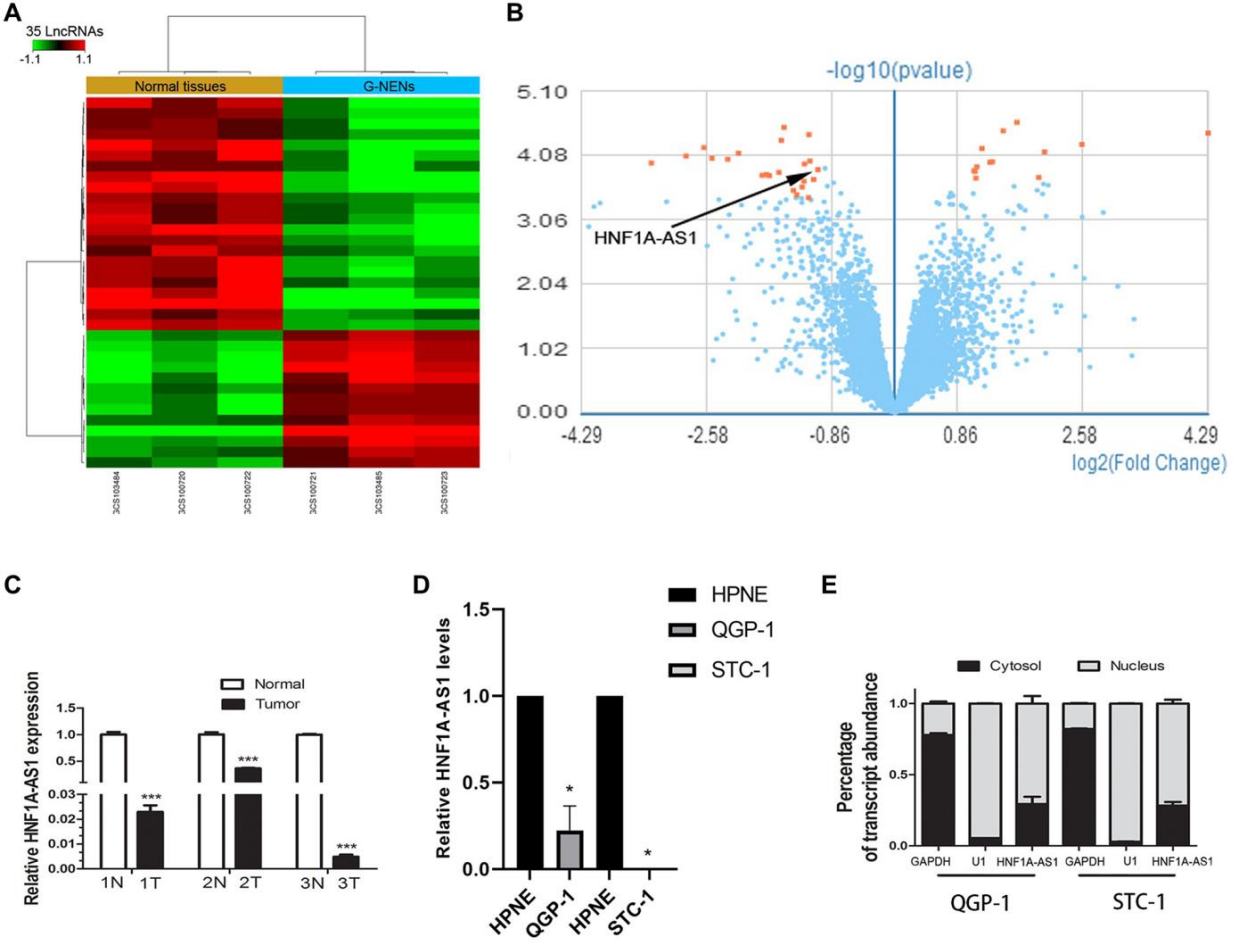
**HNF1A-AS1 was down-regulated in human G-NENs tissues and GEP-NENs cells.** (**A**) HNF1A-AS1 expression was analyzed by GeneChip assay in G-NENs tissues (*n* = 3) and peri-tumor tissues (>5 cm distant from cancer tissues) (*n* = 3). (**B**–**C**) HNF1A-AS1 was decreased in these three G-NENs tissues than in peri-tumor tissues. (**D**) HNF1A-AS1 expression was decreased in GEP-NENs cells than in human normal pancreatic cells HPNE as analyzed by qRT-PCR. (**E**) Isolation of nuclear and cytoplasmic fractions in QGP-1 and STC-1 cells suggested that HNF1A-AS1 was mainly localized in the nucleus. ^*^p < 0.05, ^**^p < 0.01.

Human GeneChip array indicated 13 up-regulated lncRNAs and 22 down-regulated lncRNAs in gastric NENs (G-NENs) and peri-tumor tissues (Figure 1A, 1B). HNF1A-AS1 was decreased in these three gastric NENs tissues (mean fold change 2.07, Figure 1B). HNF1A-AS1 was down-regulated in tumor tissues than normal peri-tumorous tissues (*p* < 0.01, Figure 1C). It was also down-regulated in GEP-NENs cells compared with normal pancreatic HPNE cell by qRT-PCR (*p* < 0.05, Figure 1D). HNF1A-AS1 was mainly localized in the nucleus of QGP-1 and STC-1 cells (Figure 1E).

### HNF1A-AS1 suppressed cell proliferation, migration and invasion *in vitro*

**Figure 2:**
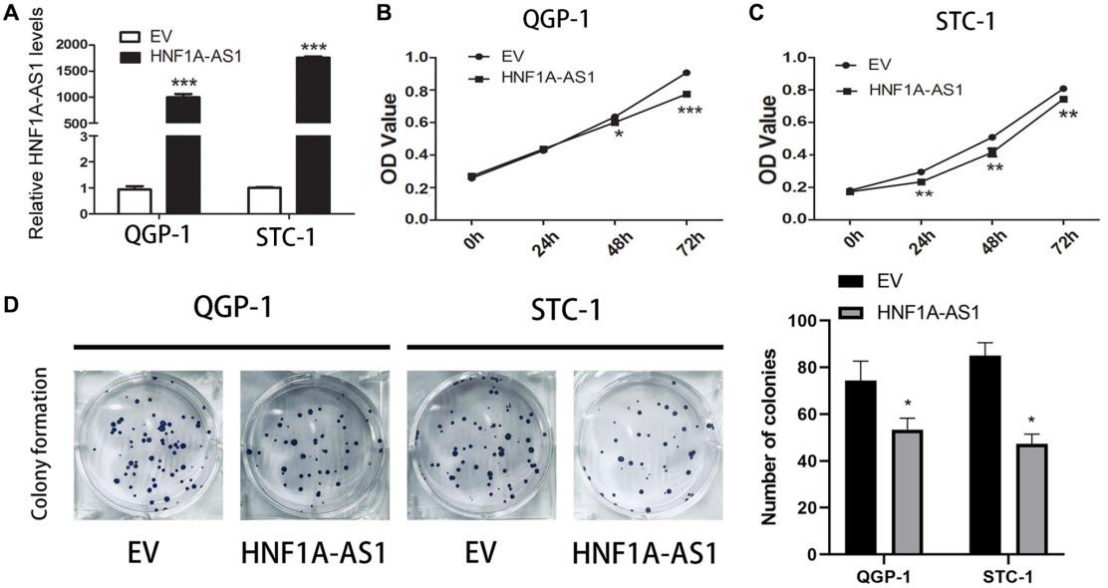
**Over-expression of HNF1A-AS1 suppressed cell viability.** (**A**) The transfection rate of HNF1A-AS1 plasmids was validated by qRT-PCR. (**B**, **C**) CCK8 was performed to analyze the growth rate of GEP-NENs cells transfected with HNF1A-AS1 over-expression plasmid or control (EV). (**D**) The colony formation ability of GEP-NENs cells transfected with HNF1A-AS1 or EV was determined.^*^p < 0.05, ^**^p < 0.01.

The expression of HNF1A-AS1 was higher with over-expression plasmid (Figure 2A). HNF1A-AS1 over-expression decreased cellular viability and attenuated the colony numbers (*p* < 0.01, Figure 2B–2D).

**Figure 3:**
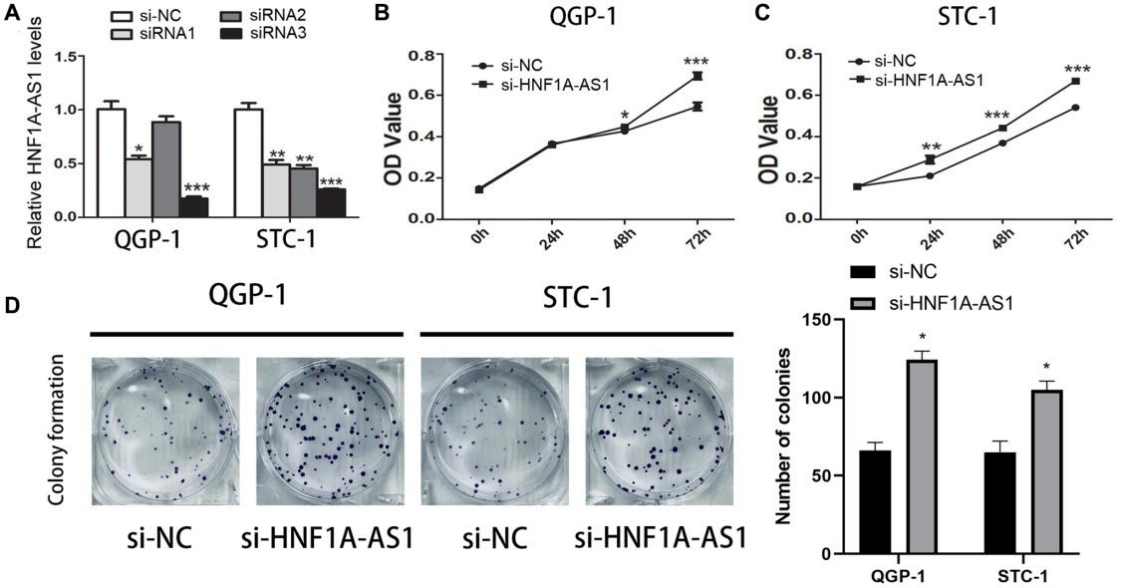
**Knockdown of HNF1A-AS1 promoted cell viability.** (**A**) qRT-PCR was performed to detect the best efficiency of siRNA-HNF1A-AS1-1, siRNA-HNF1A-AS1-2 and siRNA-HNF1A-AS1-3. Among them, siRNA-HNF1A-AS1-3 showed the best efficiency in both two cell lines. (**B**–**C**) CCK8 revealed that knockdown of HNF1A-AS1 induced increase of cell viability in both two cell lines. (**D**) The colony formation ability of GEP-NENs cells transfected with si-HNF1A-AS1 or negative control (si-NC) was determined. ^*^p < 0.05, ^**^p < 0.01.

The siRNA-HNF1A-AS1-3 showed the best efficiency in both QGP-1 and STC-1 cells (Figure 3A). HNF1A-AS1 knockdown up-regulated cell viability (*p* < 0.01, Figure 3B, 3C) and colony numbers (*p* < 0.05, Figure 3D).

### HNF1A-AS1 inhibited GEP-NENs tumorigenesis *in vivo*

**Figure 4:**
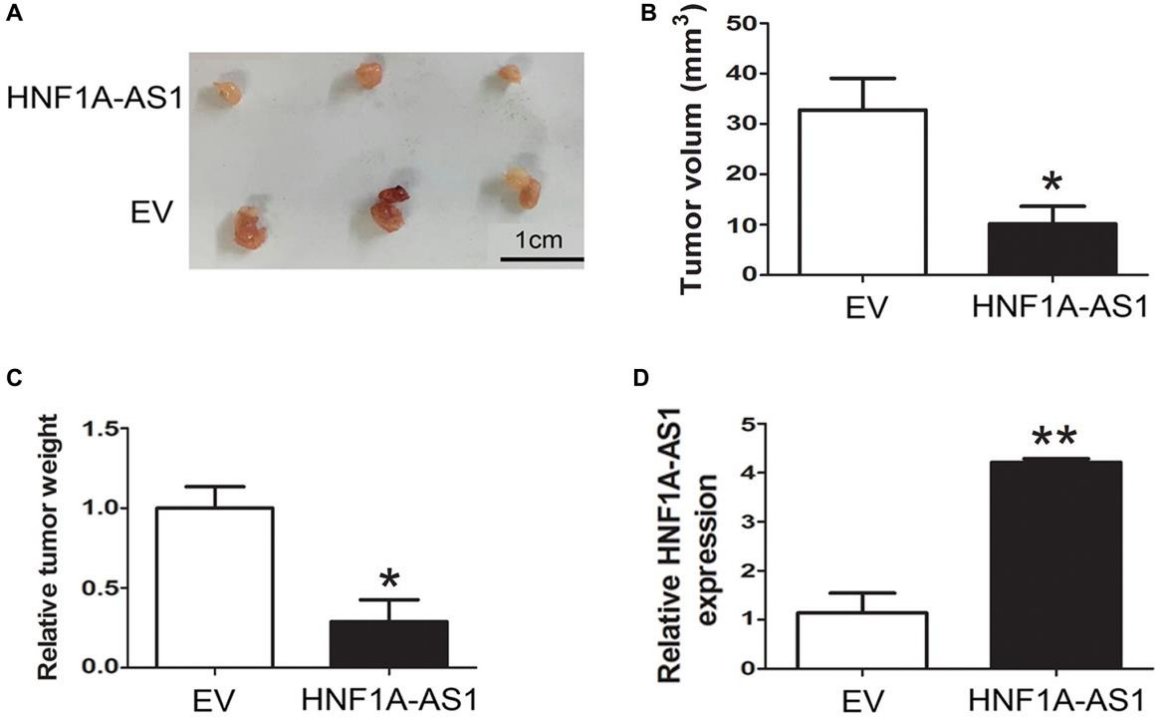
**HNF1A-AS1 inhibited GEP-NENs tumorigenesis *in vivo*.** (**A**) The HNF1A-AS1 over-expression group or EV group were used for tumorigenesis assay. Five weeks later, the mice were killed and the tumor nodules were harvested. (**B**–**C**) Tumor growth curves after subcutaneous injection of QGP-1 cells transfected with HNF1A-AS1 over-expression plasmid or EV were shown. The tumor volumes and weights were measured every 7 days after inoculation. (**D**) qRT-PCR was performed to validate the higher level of HNF1A-AS1 in over-expression group than in EV group. ^*^p < 0.05, ^**^p < 0.01.

The QGP-1 cells transfected with HNF1A-AS1 over-expression plasmid were subcutaneously inoculated into nude mice (*n* = 6 mice per group). At 49 days after injection, the tumors formed in HNF1A-AS1 over-expression group were smaller than those in control group (Figure 4A). The average tumor volume and weight were decreased either (*p* < 0.05, Figure 4B, 4C). HNF1A-AS1 expression in over-expression group was much higher than control group (*p* < 0.01, Figure 4D).

### TCF3 was the potential transcription factor of HNF1A-AS1

**Figure 5:**
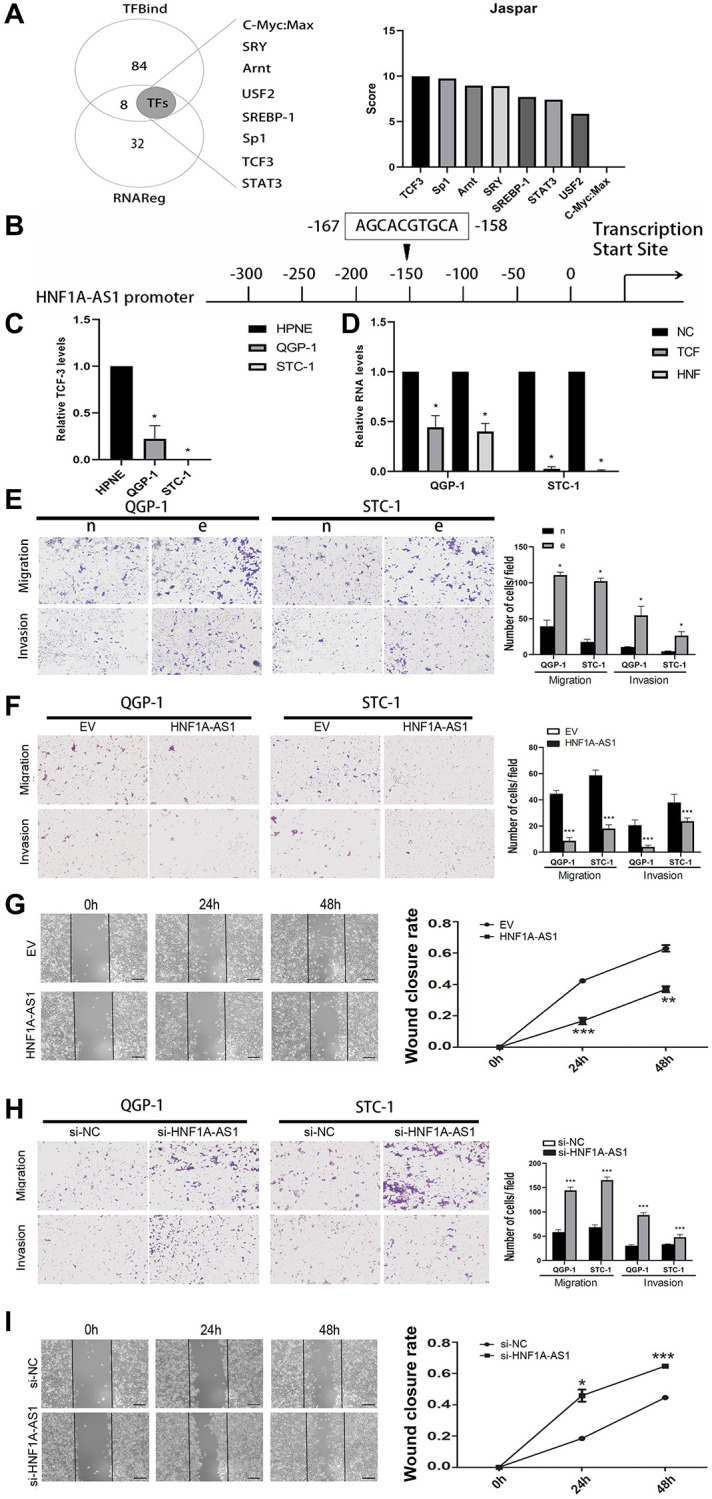
**TCF3 down-regulated HNF1A-AS1 and promoted cell migration and invasion.** (**A**) The potential transcription factors of HNF1A-AS1 were predicted by TFbind and RNAreg respectively. The score of each transcription factor was ranked by JASPAR. (**B**) JASPAR was used to detect the putative binding sites between TCF3 and HNF1A-AS1. (**C**) qRT-PCR was performed to analyze the level of HNF1A-AS1 in HPNE cell compared with in GEP-NENs cell. (**D**) Knockdown of TCF3 resulted in decrease of HNF1A-AS1 in GEP-NENs cells. (**E**) Transwell was performed to investigate the effect of TCF3 knockdown (group e) on GEP-NENs cells migration and invasion. (**F**–**G**) Over-expression of HNF1A-AS1 down-regulated cell migration and invasion by transwell and wound healing assay. (**H**–**I**) Knockdown of HNF1A-AS1 increased cell migration and invasion by transwell and wound healing assay. ^*^p < 0.05, ^**^p < 0.01.

We took intersection between 92 potential transcription factors from TFbind and 40 upstream genes from RNAreg. Totally of 8 potential transcription factors probably binding to HNF1A-AS1 were selected. We analyzed the promoter region of HNF1A-AS1 to predict score of these potential transcription factors and detect the binding sites with JASPAR program (Figure 5A). TCF3 could potentially bind to HNF1A-AS1 and the putative binding sites were confirmed (˗158 to ˗167bp, AGCACGTGCA, Figure 5B). TCF3 was decreased in GEP-NENs cells compared with normal cells by qRT-PCR (*p* < 0.05, Figure 5C). TCF3 knockdown with lentivirus down-regulated HNF1A-AS1 level (*p* < 0.05, Figure 5D).

Silencing of TCF-3 promoted cellular migration and invasion (*p* < 0.05, Figure 5E). Over-expression of HNF1A-AS1 attenuated cellular migration, invasion and wound closure (Figure 5F, 5G). On the contrary, HNF1A-AS1 knockdown had the opposite effects (Figure 5H, 5I).

### HNF1A-AS1 inhibited EMT in GEP-NENs cells

**Figure 6:**
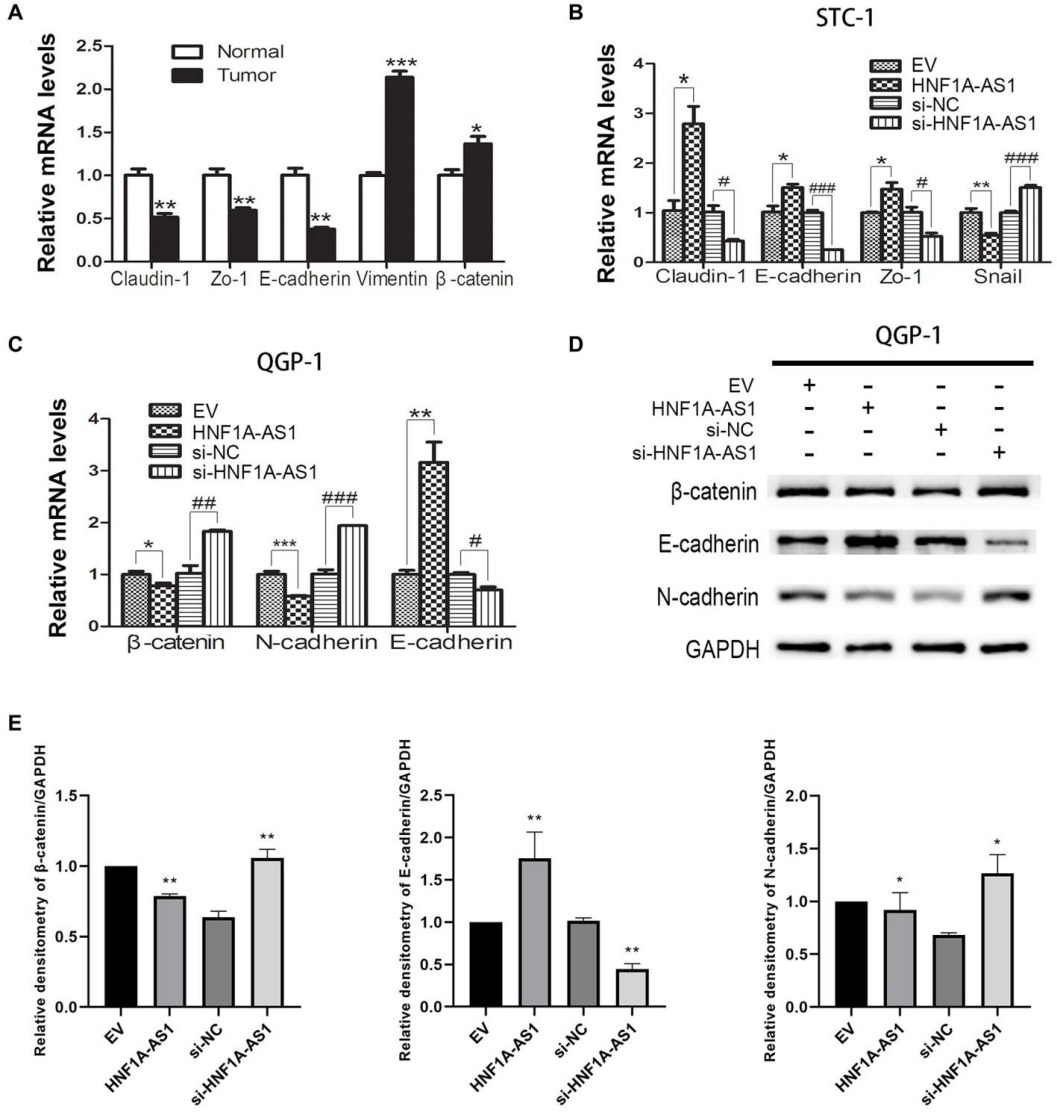
**HNF1A-AS1 inhibited EMT process in GEP-NENs cells.** (**A**) qRT-PCR was performed in G-NENs tissues to detect the EMT markers. (**B**–**C**) qRT-PCR was performed in GEP-NENs cells to detect the EMT markers. (**D**–**E**) Western blot was used to validate the level of EMT markers in GEP-NENs cells. ^*^p < 0.05, ^**^p < 0.01.

In gastric NENs tumor tissues, the epithelial markers Claudin-1, Zo-1 and E-cadherin were down-regulated, while the mesenchymal markers Vimentin and β-catenin were up-regulated as compared to non-tumorous tissues (*p* < 0.05, Figure 6A). In STC-1 cells, over-expression of HNF1A-AS1 led to up-regulation of E-cadherin, Claudin-1 and Zo-1 mRNA and down-regulation of Snail mRNA. HNF1A-AS1 knockdown resulted in adverse effects (*p* < 0.05, Figure 6B). In QGP-1 cells, over-expression of HNF1A-AS1 resulted in decrease of β-catenin, N-cadherin mRNA and increase of E-cadherin mRNA. HNF1A-AS1 knockdown induced adverse effects (*p* < 0.05, Figure 6C–6E).

### HNF1A-AS1 suppressed cellular invasion via targeting oncostatin M (OSM)

**Figure 7:**
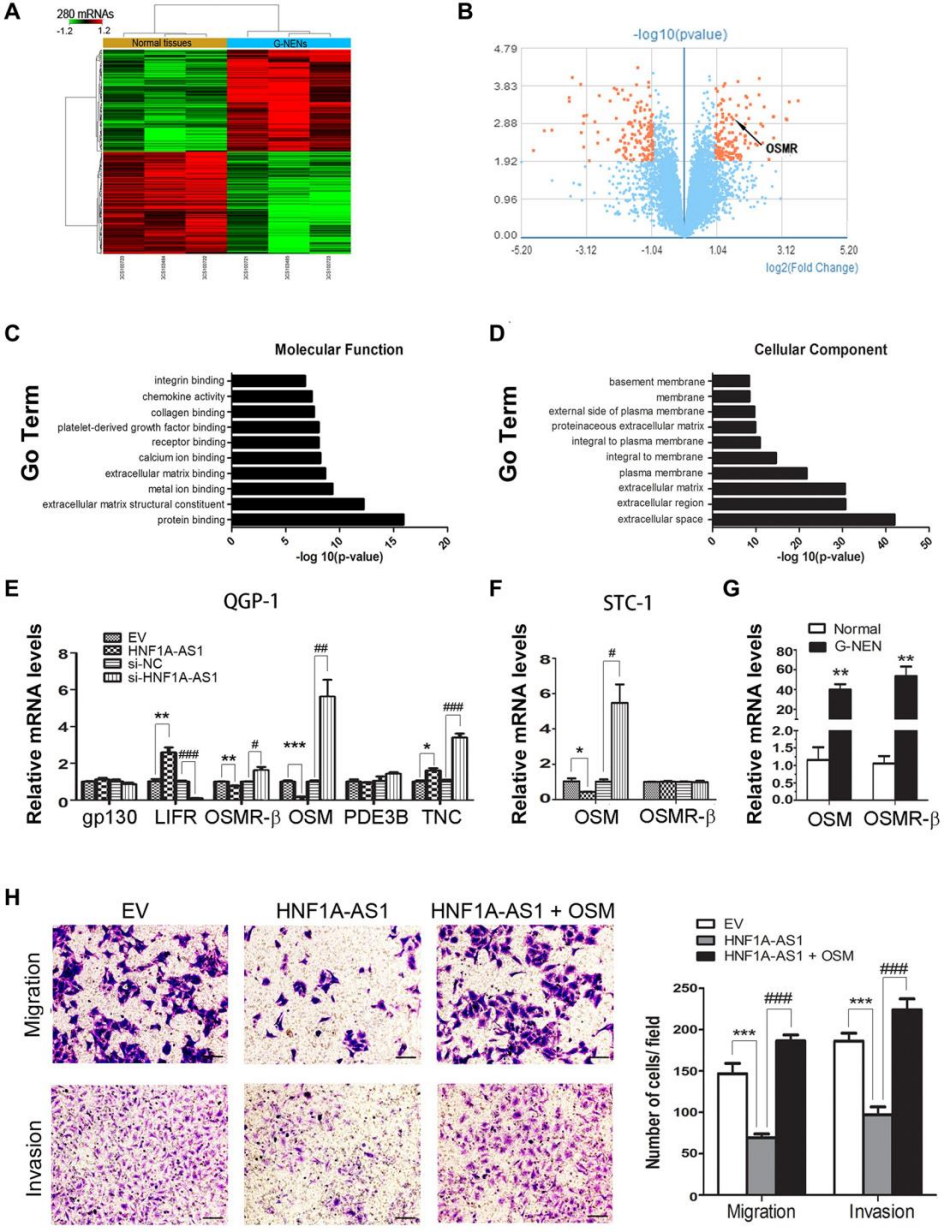
**HNF1A-AS1 inhibited cellular invasion targeting OSM.** (**A**–**B**) Totally of 280 discrepant coding genes were identified by GeneChip array analysis. (**C**) Receptor binging was found as one of the top ten GO terms in the molecular function analysis. (**D**) In cellular component analysis, extracellular space was the most represented component. (**E**) OSM expression was the most discrepant gene following dys-regulation of HNF1A-AS1 in QGP-1 cells by qRT-PCR. (**F**–**G**) OSMR-β and OSM expression was tested in STC-1 cells and human samples by qRT-PCR. (**H**) Transwell was performed to analyze the effect of OSM on cell migration and invasion. ^*^p < 0.05, ^**^p < 0.01.

Through GeneChip array analysis, we identified 280 discrepant coding genes (*p* < 0.05, Fold change >2, 140 up-regulated and 140 down-regulated), as presented in the heat map and volcano plot (Figure 7A, 7B). The GO analysis was performed based on these 280 differential genes (*p* = 0.05, FDR = 0.05). We found that receptor binging was one of the top ten GO terms in molecular function analysis (Figure 7C). In cellular component analysis, extracellular space was the most represented component (Figure 7D). Then, we chose OSM, phosphodiesterase 3B (PDE3B), Tenascin C (TNC) from these 280 discrepant genes, which had been reported as ligands to gp130, leukemia inhibiting factor receptor (LIFR), Receptor beta (OSMR-β). Interestingly, OSM expression presented the most discrepant following dys-regulation of HNF1A-AS1. HNF1A-AS1 failed to alter OSMR-β expression (*p* < 0.05, Figure 7E, 7F). We evaluated OSMR-β and OSM expression in human samples. Both OSMR-β and OSM mRNA levels were increased in gastric NENs tissues (*p* < 0.01, Figure 7G). At last, we added 50 ng/ml of OSM to STC-1 cells and found that it could reverse HNF1A-AS1-induced decrease of migration and invasion (*p* < 0.01, Figure 7H).

### TGFβ signaling was essential for HNF1A-AS1 mediated cellular migration and invasion

**Figure 8:**
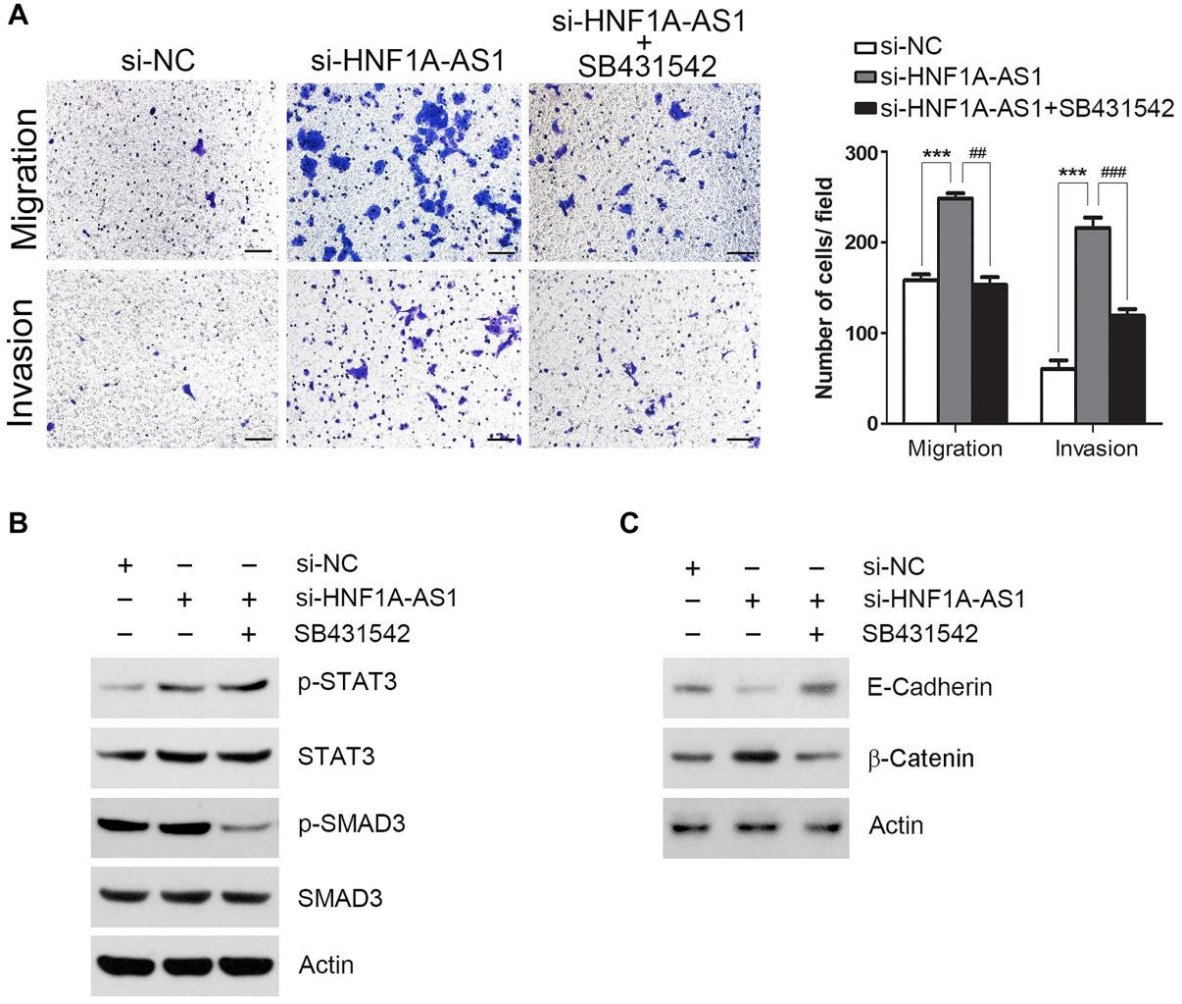
**TGFβ signaling was essential for HNF1A-AS1 mediated cell migration and invasion.** (**A**) Transwell was performed to analyze the effect of SB431542 on TGFβ signaling pathway. (**B**) TGFβ signaling transduction by si-HNF1A-AS1 and/or SB431542 was validated by western blot. (**C**) Western blot was used to analyze the effect of SB431542 on EMT markers after knockdown of HNF1A-AS1. ^*^p < 0.05, ^**^p < 0.01.

Next, we employed SB431542 (10 μM) to block TGFβ signaling pathway. SB431542 treatment reversed the cellular migration and invasion induced by HNF1A-AS1 knockdown (*p* < 0.01, Figure 8A). HNF1A-AS1 knockdown resulted in an up-regulated phosphorylated signal transducers and activators of transcription 3 (STAT3) but not Sekelsky mothers against dpp Homolog 3 (SMAD3). SB431542 inhibited phosphorylation of SMAD3, but not p-STAT3 (Figure 8B). HNF1A-AS1 knockdown decreased E-cadherin and enhanced β-catenin expression. These changes induced by HNF1A-AS1 knockdown were reversed by SB431542 (Figure 8C).

## DISCUSSION

LncRNAs exert important functions in various biological processes such as cell differentiation, proliferation and apoptosis [27, 28]. By far, the role of lncRNA HNF1A-AS1 in GEP-NENs development has not been clarified.

In this study, we found that HNF1A-AS1 level in gastric NENs tissues was decreased with GeneChip assay. HNF1A-AS1 over-expression could suppress proliferation, migration and invasion *in vitro* and *in vivo*. These effects induced by HNF1A-AS1 might be dependent on induction of EMT.

Transcription factors could bind to lncRNAs to regulate the progression of tumorigenesis and metastasis [29, 30]. We found that TCF-3 was upstream transcription factor to regulate HNF1A-AS1 transcription. Knockdown of TCF3 decreased the expression of HNF1A-AS1 and promoted cellular migration and invasion. TCF-3 had been proven to take part in human cancer development and progression [31–33]. HNF1A-AS1 enhanced cell proliferation and metastasis in osteosarcoma through activation of the Wnt/β-catenin signaling pathway [34]. In the present study, we found that knockdown of TCF3 could bind to the specific site of HNF1A-AS1 promoter region which induced down-regulation of HNF1A-AS1 and promoted tumorigenesis and metastasis in GEP-NENs.

OSM had been considered as a pleiotropic cytokine. It exerted its biological function by binding to two different OSM receptor complexes, OSMR-β and LIFR [35]. Aberrant expression of OSM promoted cellular invasion and induced mesenchymal phenotype in many solid tumors including osteosarcoma, gliomas and breast cancer [36–39]. We found that HNF1A-AS1 could inhibit OSMR-β and OSM expression. Previous studies had reported that OSM promoted cancer cell plasticity through cooperation with STAT3-SMAD3 signaling [40]. It has also been reported as a novel inhibitor of TGFβ-induced matricellular protein expression [41–43]. In our study, we discovered that TGFβ signaling was essential for cellular invasion induced by HNF1A-AS1/OSMR.

In summary, HNF1A-AS1 was down-regulated in GEP-NENs tissues. TCF3-mediated HNF1A-AS1 inhibited cellular proliferation and invasion via OSM/TGFβ signaling. HNF1A-AS1 may be served as a potential target for further diagnosis and treatment in human GEP-NENs.

## MATERIALS AND METHODS

### Tissue specimens and cell lines

The gastric NENs tissues and peri-cancerous tissues (>5 cm distant from cancer tissues) were obtained from three patients who underwent surgical resection at the First Affiliated Hospital of Nanjing Medical University. All patients did not receive any local or systemic treatment before surgery. All experiments were approved by the Research Ethics Committee of Nanjing Medical University. Written informed consent was obtained from all participants.

The human pancreatic NENs derived QGP-1 cell line was registered with the JCRB cell bank (JCRB0183). The STC-1 cell line was purchased from the ATCC (CRL-3254). HPNE (Human Pancreatic Nestin-Expressing ductal cells) was also obtained from the ATCC (CBP60857). QGP-1 cells and HPNE cells were cultured in RPMI-1640 (Gibco, Carlsbad, CA, USA) containing 10% fetal bovine serum (FBS; Gibco) at 37°C, 5% CO_2_. STC-1 cells were cultured in Dulbecco’s Modified Eagle Medium (Gibco) supplemented with 10% FBS.

### Gene expression profiles and data analysis

Total RNA was extracted and cDNAs were prepared according to the standard Following labeling, 5.5 μg of cDNA were hybridized for 16 h at 45°C on GeneChip Human Transcriptome Array 2.0. The arrays were scanned using the GeneChip^®^ Scanner 3000 7G. Raw data were analyzed using Robust Multiarray Analysis (RMA) with a log base 2 (log_2_) transformation. The threshold was fold change > 2 and *p*-value < 0.05.

### Construction of plasmids and cell transfection

Plasmids including small interfering RNA targeting HNF1A-AS1 (si-HNF1A-AS1) and HNF1A-AS1 over-expression plasmid (oe-HNF1A-AS1) were purchased from RiboBio (Guangzhou, China). Three siRNA targeting HNF1A-AS1 (si-HNF1A-AS1-1, si-HNF1A-AS1-2 and si-HNF1A-AS1-3) and scrambled negative control (si-NC) were designed and synthesized by RiboBio (Guangzhou, China). The siRNA sequences were as follows: siRNA1 - CCCTCCATCTAACATTCAA, siRNA2 - GCAGCTGTTTGCAAGACTA, and siRNA3 - CCCTCATCCTGGCATTCAA. Full-length coding sequence for HNF1A-AS1 was amplified into pcDNA 3.1(+) vector (Life technologies) according to the manufacturer’s instructions.

### Construction of TCF3 knockdown lentivirus

The short hairpin RNA (shRNA) sequence targeting TCF3 (CCGGCTCCTAATGTCAACCGAGAACTCGAGTTCTCGGTTGACATTAGGAGCTTTTT) was obtained from GeneChem, Inc., Recombinant lentiviral vectors were constructed according to previous studies. The transfection efficiency of shTCF3 was determined using reverse transcription-quantitative (qRT-)PCR and western blot analysis after 72 h.

### qRT-PCR

RNA was extracted from each fraction using TriPure Isolation Reagent (Roche, USA). The separation of nuclear and cytosolic fractions was conducted using the NE-PER Nuclear and Cytoplasmic Extraction Reagents kit (Thermo Fisher Scientific) according to the manufacturer’s protocol. cDNA was synthesized using a Reverse Transcription Kit (Takara, Dalian, China). qRT-PCR analysis was conducted with Essential DNA Green Master (Roche, USA). The results were normalized to GAPDH. Primers for amplification of HNF1A-AS1 were designed as follows: Forward: 5′-TCAAGAAATGGTGGCTAT-3′, Reverse: 5′-GCTCTGAGACTGGCTGAA-3′.

### Cell viability and proliferation assay

Cell viability was assessed by CCK-8 assay. Cells were seeded at density of 3000 per 100 μl medium per well in 96 well culture plates. At different time points after transfection, 10 μl of CCK-8 (Dojindo) reagent was added to wells and further incubated for 4 h at 37°C. The absorbance was measured at 450 nm after 1–4 h.

Suspended pre-treated cells were seeded in culture dishes at a density of 300 cells per well and incubated at 37°C until visible colonies emerging (2–3 weeks). Cells were fixed and stained before counting the colonies.

### Cellular migration and invasion assays

Pre-treated cells were seeded into the upper Transwell™ chambers 36 h after transfection for migration assays (8 μm pore size, Millipore) and invasion assays with the Matrigel-coated (BD, Franklin Lakes, NJ, USA) filters in 24-well plates. Cells in the upper chambers were cultured with 300ul DMEM/F12 or RPMI-1640 medium without FBS. The lower chambers were DMEM/F12 or RPMI 1640 medium containing 10% FBS. Then, cells that passed through the filters were stained and photographed from random fields.

At 24 h after transfection, wound healing assay was performed using Ibidi cell migration technology (Ibidi, Martinsried, Germany). Cells were seeded at density of 3–7 × 10^5^/ml per well and scraped two perpendicular straight lines. Cells were photographed at different time points. The migration rates were shown as the percentage of area reduction of wound closure by ImageJ.

### Protein extraction and western blotting

Cell proteins were extracted from cells as usual. Samples from cell lysates were resolved by SDS-PAGE and then transferred to polyvinylidene fluoride (PVDF) membrane and incubated with specific antibodies against E-Cadherin, N-cadherin, β-catenin, GAPDH and other markers (Cell Signaling Technology, MA, USA). The ECL chromogenic substrate was used to detect specific bands.

### Bioinformatics analysis

The HNF1A-AS1 sequence was downloaded from UCSC Genome Browser (http://genome.ucsc.edu/), from which the 2,000-bp transcription start site (TSS) upstream sequence was extracted. TFbind (http://tfbind.hgc.jp/) and RNAreg were used to detect the potential transcription factors of HNF1A-AS1. To identify the putative transcription factors and their binding sites, promoter sequence of HNF1A-AS1 was submitted to the JASPAR program (http://jaspar.genereg.net/).

### Tumor formation assay

All animals were approved by the Committee on the Ethics of Animal Experiments of the Nanjing Medical University. The athymic male BALB/c nude mice (4 weeks old) were obtained from the Shanghai Laboratory Animals Center of the Chinese Academy of Sciences (Shanghai, China). A total of 5 × 10^6^ QGP-1 cells transfected with pcDNA3.1-HNF1A-AS1 or pcDNA3.1 empty vector (EV) were subcutaneously injected into male BALB/c nude mice. Seven weeks after cell injection, the mice were sacrificed. The volume and weight of each excised subcutaneous tumor was measured. The tumor volumes were calculated by the following formula: 0.5 × length × width^2^.

### Statistical analysis

All the experiments were carried out at least three times independently. Bars shown represented mean ± SEM. The differences between independent groups were analyzed by Student′s *t*-test using SPSS software, the correlation between TCF-3 levels and HNF1A-AS1 was assessed using Spearman’s correlation coefficient, and a *p* value < 0.05 was considered to be significant.
